# Divergence in the evolution of Paleolithic symbolic and technological systems: The shining bull and engraved tablets of Rocher de l'Impératrice

**DOI:** 10.1371/journal.pone.0173037

**Published:** 2017-03-03

**Authors:** Nicolas Naudinot, Camille Bourdier, Marine Laforge, Céline Paris, Ludovic Bellot-Gurlet, Sylvie Beyries, Isabelle Thery-Parisot, Michel Le Goffic

**Affiliations:** 1 University Côte d’Azur, CNRS, CEPAM, UMR, Nice, France; 2 University Toulouse II Jean Jaurès, TRACES UMR, Toulouse, France; 3 EVEHA, Études et valorisations archéologiques, Rennes, France; 4 UMR 6554 LETG Brest-Géomer, Littoral, Environnement, Télédétection et Géomatique, Institut Universitaire Européen de la Mer, Plouzané, France; 5 Sorbonne Université, UPMC Université Paris 6, MONARIS UMR, UPMC, CNRS, Université Pierre et Marie Curie Paris 6, Paris, France; 6 UMR 6566 CReAAH, Rennes, France; Université de Poitiers, FRANCE

## Abstract

The development of the Azilian in Western Europe 14,000 years ago is considered a “revolution” in Upper Paleolithic Archaeology. One of the main elements of this rapid social restructuring is the abandonment of naturalistic figurative art on portable pieces or on cave walls in the Magdalenian in favor of abstract expression on small pebbles. Recent work shows that the transformation of human societies between the Magdalenian and the Azilian was more gradual. The discovery of a new Early Azilian site with decorated stones in France supports this hypothesis. While major changes in stone tool technology between the Magdalenian and Azilian clearly mark important adaptive changes, the discovery of 45 engraved schist tablets from archaeological layers at Le Rocher de l’Impératrice attests to iconographic continuity together with special valorization of aurochs as shown by a “shining” bull depiction. This evidence suggests that some cultural features such as iconography may lag far behind technological changes. We also argue that eventual change in symbolic expression, which includes the later disappearance of figurative art, provides new insight into the probable restructuring of the societies.

## Introduction

The Azilian is a culture of the European Upper Paleolithic. It appears at the end of the Magdalenian around 14,000 years ago and precedes the first Mesolithic hunter-gatherers in the early Holocene. This period is critical to the study of cultural evolution as it is characterized by a major restructuring of hunter-gatherer societies in terms of technology (a decrease in stone and bone tool standardization and a simplification of manufacturing processes), settlement (with changes in mobility patterns), and art, with the development of a unique abstract graphic production that contrasts with the previous Paleolithic but also the final Paleolithic “Laborian” figurative iconography (e.g., Lascaux, Altamira) [1–5]. The role that rapid warming and climatic instability of the Azilian period played in shaping techno-economic and symbolic spheres is debated among scholars [6–8]. Based on genetic studies, some other scholars also emphasize the potential role played by the migration of new populations coming from Eastern Europe and the Near East around 14,000 BP [9].

The Azilian has long been considered an abrupt event, a “revolution” in the Upper Paleolithic [10]. Recent work shows that the development of this culture was not sudden but progressive [6, 11–12]. This global process of cultural change began during the GI-1e/d (end of the Bølling) with the Early Azilian (EA) [6, 8, 12–14]. However, the Azilian probably finds its roots at the end of the Magdalenian during the GS-2b-a (Oldest Dryas) [6, 15]. The paroxysm of this process is the so-called Late Azilian (LA), probably lasting to the first half of GS-1 (Younger Dryas) [11].

A new EA rock shelter in the western extremity of Brittany provides critical data to investigate the tempo of technological and symbolic change during the Azilian. The association of a lithic industry with a rich artistic assemblage of 45 engraved (and sometimes charcoaled) schist stones suggests a clear arrhythmia between symbolic production and technological adaptations. Here the possible techno-economic adaptations to climatic changes appear to have had no direct influence on the symbolic and perhaps spiritual universe of the first “Azilian” people who perpetuated an age-old tradition.

## 1. Methods

### 1.1. Field work

The Rocher de l’Impératrice has been excavated every summer since 2013 and the excavation is still underway (The excavation of the site was authorized by the French Ministry of Culture. French prefectural decrees: 2013–007, 2014–031, 2015–018, and 2016–032). Because of the position of the site at the foot of a quartzite cliff in the middle of a forest, no mechanic equipment is used to excavate the site. Because of the presence of archaeological material, and especially engraved schist slabs, in the entire stratigraphy, no shovels or picks are used, only trowels and precision excavation tools. All artifacts discovered during the excavation are recorded in three dimensions with a total station. All the sediments are water screened (1.5 mm mesh) and all the lithic elements, charcoals fragments, and schist fragments are collected during this procedure. In total, 4659 lithic artefacts have been collected since 2013. Among these remains are 45 engraved schist tablets. The entire collection is temporarily stored at the UMR 7264 CEPAM CNRS at the Université Côte d’Azur in Nice (France) for the time of the study and will be store in an official Ministry of Culture repository in Brittany at the end of the program.

### 1.2. Technological study of the lithic assemblage

The technological study of the lithic assemblage has been developed following the French methodology of *Technologie lithique*. This method addresses lithic tools as the result of a production process rather than as just a cultural object (see among others [16–19]). This approach considers each lithic artifact in terms of information about the objective of production and the methods. All the information from raw material procurement to the discard of tools is considered, including production methods, curation strategies, and tool function. This method constitutes a particularly efficient tool to investigate cultural changes, since each technical culture develops particular technologies and strategies, and in hunter-gatherer socio-economic organization, since this approach allows studying spatial and temporal organization of the production (*chaine opératoire* segmentation).

### 1.3. Preliminary study of the engraved slabs

Only a preliminary study of the engraved stones has been conducted so far. Indeed most of the pieces have sediment deposits and crusts on their surfaces which make the identification of the engravings and the deciphering of the lines difficult. A meticulous cleaning will be essential to allow an exhaustive analysis of the etchings technology, forms, and superpositions or “stratigraphy” of the etchings. However, after residues of pigment were found within the engravings on one piece (317 –see below), the choice was made not to clean the other fragments. Moreover, refitting work is ongoing, for new engraved fragments are unearthed each year at the site. Hence, only the main motifs from the two major pieces are discussed here. Although preliminary, analysis of these images have great archaeological significance. And since the deposits on these two stones are restricted, few new motifs are expected to come out of the analytical tracings. “Reading sketches” were made from a photo-mosaic created with pictures taken under different lighting conditions, and according to the classic tracing method used in the study of mobile art.

A first technical study was conducted on the most striking decorated set of the series: an auroch’s head surrounded by radiating lines made of very wide and deep engraving (fragment 317 side A). A fine analysis of the engraving (techniques, chronology of the incisions, dyes…) requires a proper cleaning of the tablets. The analysis of the morphology of the incisions (edges and bottom), the regularity and precision of the lines can only be done after a precise observation with selected optical instruments on surfaces cleaned of any sediment. A first light cleaning has pointed out the presence of black pigments in the wide and deep engravings; hence we have opted for a long but non-aggressive protocol to clean the engravings and highlight these deposits. The tablets were cleaned under a microscope for a more precise view of the surfaces. If adhering sediment had chipped, a light lever made with a wooden toothpick removed it. When the sediment was too hard, it was softened with distilled water and then a small strip of fiber-free paper was placed on the soaked area and the sediment removed by transfer. Some areas were left uncleaned to preserve trapped dyes for future analysis. These areas have been selected out of the etching intersections, in order to allow the most complete possible decoding of the chronology of the engraving process.

### 1.4. Analytical characterization of the engraving black pigment

Pigment characterization was achieved non-invasively by Raman spectroscopy using a Labram HR800 (Horiba Jobin Yvon) spectrometer and the blue 458nm line from an Argon Laser (Innova 90C-6 from Coherent). A microscope allows the visualisation of the analysed area and the interfacing of the micro-measurement with the sample through the focusing of the Laser and the collection of the Raman scattering. Long working distance 50x and 100x objectives (Olympus) were used, allowing an area of analysis of about 2 and 1 micrometers, respectively. The Laser power at the sample is set around 100 μW in order to respect sample integrity. The artifact was directly positioned on the microscope stage to perform the non-contact analyses. Such analyses allow the structural identification of the material used as a pigment in order to determine its nature.

## 2. Le Rocher de l’Imperatrice rock shelter

**Fig 1:**
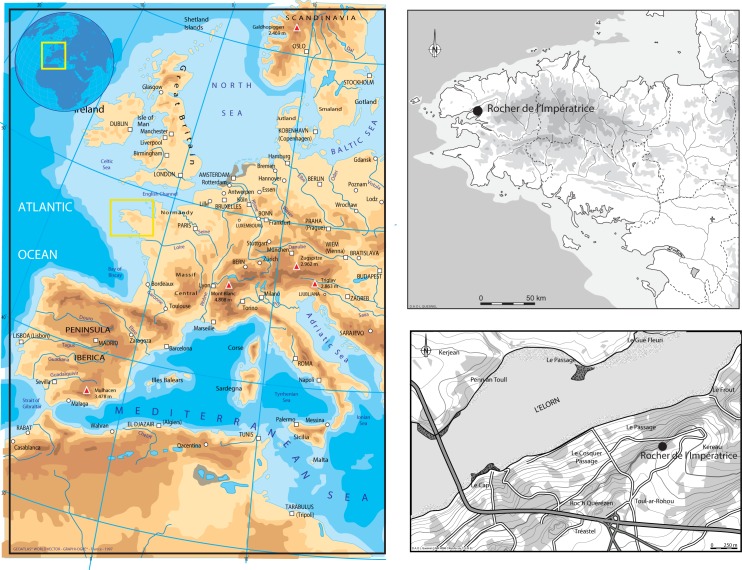
Location of Le Rocher de l’Impératrice rock-shelter (Europe map from Geoatlas, Brittany and local maps L. Quesnel (CNRS)).

Le Rocher de l’Impératrice is a small rock-shelter approximately 10 m long, 3 m deep and 2 m high located near Plougastel-Daoulas at the western extremity of Brittany (France). The shelter is at the foot of a 50 m high quartzite cliff dominating the Brest roadstead (Fig 1). The site sits about 50 m a.s.l on a southern steep slope overlying Brioverian shale bedrock. The steep topography is covered by silty-clayey solifluction deposits rich in shale flags.

**Fig 2:**
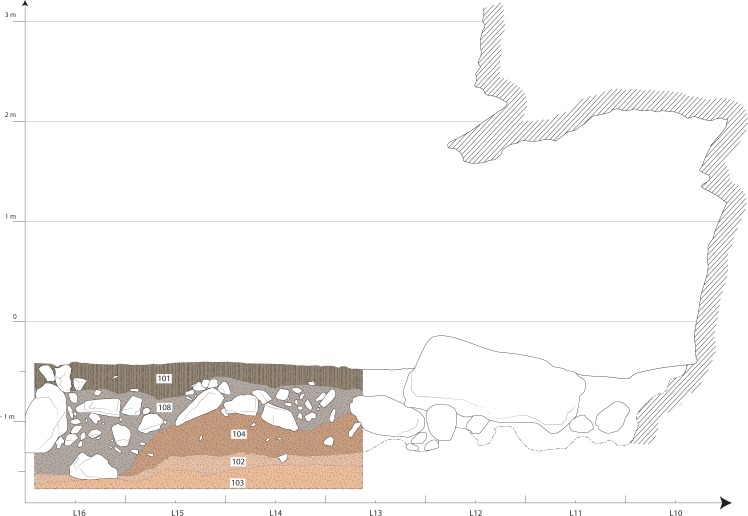
Summary cross section of the central area of the rock-shelter. US- 101: Humic horizon on silty-clayey colluvium and modern anthropogenic filling. US-108: Scree of quartzite boulders in silty-clayey colluvium. US-104: Strongly developed colluvium (Bt horizon). US-102: Solifluction layer made of reworked lœss and small cryoclastic quartzite boulders and gravels. US- 103: Lightly reworked lœss (M. Laforge & S. Sorin).

Because of the geomorphologic context, the stratigraphic sequence of the rock-shelter is complex. Sedimentary infilling of the shelter begins with alternating facies of lightly reworked loess and solifluction deposits (US-103) (Fig 2). US-103 is cryoturbated and was deposited by short distance solifluction or gelifluction, under periglacial conditions, possibly at the end of the Last Glacial Maximum (MIS 2) or during the beginning of Late Glacial (c. 15, 000 BP). US-103 consists of a reactivation of earlier cryoclastic rock fall and slope deposits (cliff-talus slope system) mixed with reworked aeolian deposits, due to the secondary redistribution of the debris on this slope. US-102, truncating and overlying US-103, is laminated, and made of reworked loessic matrix that includes numerous small quartzite blocks. The laminations of these solifluction deposits are reflect minor fluctuations in deposition, which was probably always under periglacial conditions with sparse vegetation [20]. This deposit may correspond to an accumulation of solifluction lobes, usually developed on 2 to 35° slopes[21]. US-102 is characterized by increased illuviation, highlighting the former laminations (Bt horizon). The observed pedogenesis may be explained by the presence of an ancient and eroded soil, developed on permafrost, during the end of LGM [22], around the Early Bølling, ca. 16, 000 BP. US-102 contains, especially in the upper part of it, most of the azilian artifacts and makes up the archaeological layer. Above these formations, several units of colluvium were laid down (US-104 and US-108). US-104 is a partially preserved colluvium, deposited by runoff, anterior to the late Neolithic recorded in US-108 and strongly pedogenetised (old Bt horizon). Above it, US-108 includes lots of plurimetric quartzite boulders that fell from the overhanging Ordovician bar, and mixed within the colluvial silty-clayey matrix. The boulder rock falls, which are a part of this infilling, are important as they locally protect the former layers from erosion. The lithic artifacts and a radiocarbon date suggest this layer was mostly deposited at the end of the Neolithic. The top unit of the stratigraphic sequence of the rock-shelter, is a strongly disturbed unit (US- 101), made of humic silty-clayey colluvium reworked by recent anthropogenic digging and filling.

Within US-102 we have discovered an unquestionable archaeological layer attributed to the EA. US-102 is preserved in most areas of the site, and occurs towards the bottom of the stratigraphic sequence. Since we know that the debris flow of US-102 unit was not reworked after the Lateglacial, Azilian populations could have lived on this unit, which is confirmed by the high concentration of artifacts within this layer. Furthermore, the identification of a roof collapse event of large quartzite boulders between the Neolithic and the Azilian favors the preservation of the Azilian-aged soil under the largest blocks.

Because the site is located in the Armorican Massif, the acidic soil does not allow for the preservation of organic materials. As for most sites within this region of France, our studies and hypotheses are primarily based on the study of lithic materials. Due to the presence of a regular blade industry produced by soft hammerstone, emblematic early Azilian bipoints (curved back points with two opposed tips), and flat retouched blades, the lithic assemblage can be assuredly attributed to the EA. These reduction techniques and artifacts characterize assemblages from other well-excavated and preserved sites in Europe that date to this time period, including the lower layer of Le Closeau [14], La grotte du Cheval [23], layers 4 sup./3B of Pont d’Ambon [24], layer 4 of Abri Murat [25], layer 3 of area 1 of La Fru [12] and layer 4 of Bois-Ragot [26]. Three radiocarbon dates at Le Rocher de l’Impératrice fall into the GI-1 (Bølling for two of them and the Bølling-Allerød transition for the other) (Table 1) and support this attribution. These radiocarbon dates fit perfectly with other assays for this period in France (Table 2). The Rocher de l’Impératrice assemblage is culturally homogeneous, especially in US-102. Most of the artifacts discovered in the disturbed units of the rockshelter are also related to this Upper Paleolithic techno-complex. Very few Mesolithic artifacts have been discovered and only a small Neolithic assemblage is present in US-108. Although several of the engraved stones were discovered in a disturbed unit of the shelter, the entire corpus can be reasonably attributed to the EA occupation(s). Indeed, the horse-aurochs theme as well as the style of the figures undoubtedly refer to the Paleolithic fine naturalistic art which completely differs from the Neolithic and Mesolithic graphic works currently known in Western Europe [27–29]. There is also no evidence of dates or artifacts related to other Upper Paleolithic period. The Rocher de l’Impératrice is one of the very few EA sites with art but no evidence of a Magdalenian occupation. The geological context and artefact assemblage allows for a discussion of the technical and symbolic changes during this period.

**Table 1:** Radiocarbon dates of Le Rocher de l’Impératrice.

Laboratory number	Material dated	US	Conventional Radiocarbon age	2 sigma calibration (cal. BP)	Remarks
Beta-415532	Charcoal (*Confer Salix*)	US-102	12460 ± 50BP	14935 to 14270	
Beta-415533	Charcoal (Pericarp)	US-102	12380 ± 50BP	14715 to 14165	
Beta-415531	Charcoal (Gymnosperm)	US-102	12060 ± 40BP	14035 to 13775	
Beta-415529	Charcoal (*Quercus f*.*c*.)	US-108 (Neolithic)	4390 ± 30BP	5045 to 4865	
Beta-415530	Charcoal (*Betula sp*.)	US-102	2120 ± 30BP	2290 to 2275 and 2155 to 2000	Intrusive

**Table 2:** Radiocarbon dates for the French Early Azilian.

Site	Layer	Laboratory code	Conventional Radiocarbon age	2 sigma calibration (cal. BP)	Reference	Remarks
Pont d'Ambon	4 sup.	Gif-3369	12840 ± 220	14068 to 12456	[24]	
Bois-Ragot	4	OxA-10334	12720 ± 100	13582 to 12768	[30]	
Abri Murat	4	GifA-92345	12620 ± 130	13392 to 12311	[31]	
Bois-Ragot	4	OxA-10333	12585 ± 75	13261 to 12498	[30]	
Le Closeau, locus 33	Intermediary	GrA-18860	12510 ± 80	13158 to 12326	[32]	
Le Closeau, locus 33	Intermediary	GrA-18815	12480 ± 70	13109 to 12303	[32]	
Bois-Ragot	4	OxA-10332	12475 ± 75	13110 to 12287	[30]	*Might be from the Magdalenian layer*. *Date on a reindeer bone*
Pont d'Ambon	3B	Ly-6433	12450 ± 70	13057 to 12256	[13]	
Abri Murat	4	Poz-27957	12430 ± 80	13043 to 12215	[13]	
Le Closeau, locus 46	Lower layer	AA-41881	12423 ± 67	12994 to 12220	[32]	
Le Closeau, locus 46	Lower layer	GrA-11665	12360 ± 90	12956 to 12121	[32]	
Le Closeau lower layer locus 46	Lower layer	GrA-18816	12350 ± 70	12835 to 12133	[32]	
Le Closeau lower layer locus 46	Lower layer	GrA-11664	12350 ± 60	12791 to 12149	[32]	
Le Closeau lower layer locus 56	Lower layer	GrA-18819	12340 ± 70	12814 to 12125	[32]	
Abri Murat	4	Poz-27958	12330 ± 80	12841 to 12095	[13]	
La Fru, aire 2	3		12300 ± 70		[33]	
Rhodes II	F5	MC-996	12300 ± 150	13045 to 11881	[34]	
La Fru, aire 2	3		12250 ± 60		[33]	
Rhodes II	F5	MC-1366	12250 ± 200	13126 to 11791	[34]	
Le Closeau, locus 46	Lower layer	AA-41882	12248 ± 66	12598 to 12003	[32]	
La Fru, aire I	3	Gr1-34354	12200 ± 50		[33]	
Rhodes II	F5	Gif-2258	12160 ± 160	12837 to 11661	[34]	
La Fru, aire I	3	Ly-134/Oxa-5264	12110 ± 110		[33]	
Pont d'Ambon	3B base	Gif-3739	12130 ± 160	12757 to 11633	[24]	
Rhodes II	F6 base	MC-997	12100 ± 150	12639 to 11621	[34]	
Le Closeau, locus 4	Lower layer	OxA-5680 (Lyon 166)	12090 ± 90	12223 to 11796	[14]	
Bange	E/G	OxA-540	12080 ± 180	12761 to 11541	[33]	
Gouy		GifA-92346	12050 ± 130	12315 to 11614	[35]	
Le Closeau, locus 4	Lower layer	OxA-6338 (Lyon 313)	12050 ± 100	12224 to 11681	[14]	
La Fru, aire I	3 base	GrA-25052	11950 ± 60	12038 to 11640	[33]	
Les Douattes, Est	4/5, F5-64	Ly-1417	11945 ± 85	12072 to 11617	[33]	
St-Thibaud-de-Couz		Ly-429	11900 ± 360	13113 to 11140	[33]	
Balma Margineda	10	Ly-4898	118701 ± 110	12035 to 11517	[36]	
La Fru, aire I	3	GrA-25080	11840 ± 60	11828 to 11540	[33]	
La Fru, aire I	3	Ly-2408	11820 ± 230	12341 to 11188	[33]	
La Fru, aire I	3	Ly-2250	11810 ± 160	12085 to 11373	[33]	
La Fru, aire I	upper 3	GrA-25054	11790 ± 60	11797 to 11528	[33]	
La Fru, aire 1	3 base	Ly-4325	11740 ± 110	11829 to 11390	[33]	
Balma Margineda	10	Ly-4896	11690 ± 90	11786 to 11393	[36]	
La Fru, aire I	3 base	Ly-2409	11680 ± 150	11871 to 11240	[33]	
Hangest III.1	Lower layer	OxA-4432 (Ly-22)	11660 ± 110	11785 to 11340	[37]	Probably older. Layer under the Allerød soil
Bois-Ragot	4	Lyon-2754 (OxA)	11640 ± 55	11630 to 11398	[30]	
Hangest III.1	Lower layer	OxA-4936 (Ly-86)	11630 ± 90	11760 to 11326	[37]	Probably older. Layer under the Allerød soil
Balma Margineda	9	Ly-5416	11600 ± 280	12162 to 10902	[36]	
Pont d'Ambon	3B base	Gif-7223	11600 ± 120	11768 to 11257	[24]	
Balma Margineda	10	Ly-5413	11560 ± 230	12018 to 11038	[36]	
Balma Margineda	10	Ly-5414	11510 ± 100	11598 to 11194	[36]	
Balma Margineda	10b	Ly-5415	11500 ± 150	11752 to 11116	[36]	

Because the lithic assemblage is limited and artifacts are restricted to the inside of the shelter with little evidence of hearth-related activities (no hearths and few burnt artifacts or charcoal) we suggest this small quartzite shelter was occupied by a small group during the EA (and probably several times by several groups). The very high proportion of retouched tools on the site (42% of the assemblage) along with only scant evidence of lithic production activities (only one core and low proportion of by-products) suggest that people probably arrived equipped with tool kits consisting of blades and prepared cores used to produce small blades designed for the creation of projectile points. Cores used to make these tools were afterwards carried out of the site. These data suggest that the site was occupied for short periods of time for specific tasks. More precisely, the focus on the manufacture of projectile points (presence of preforms and very high frequency of micro-flakes produced during the manufacture of the points) and use (30% of points with impact fractures) suggests use of the rockshelter as a hunting camp. The importance of unretouched and retouched regular blades in the assemblage could also support this hypothesis maybe suggesting butchering activities on the site. The use wear analysis of the lithic assemblage by J. Jacquier is in course and will soon bring important data on this topic. The engraved tablets, however, make this scenario more complex, suggesting not only an economic but also a symbolic function of the site. Moreover, they question the purpose of the graphic production within the economic sphere of these people, especially within their food supply. Was the iconography linked with the hunt, perhaps to ensure its success? Was it strictly symbolic, or a mere past time?

## 3. Results

### 3.1. Main characteristics of the lithic assemblage

EA sites are scarce in Western Europe and so the lithic assemblage discovered at Le Rocher de l’Impératrice is a new milestone in the perspective of understanding the shifts in technology between the Magdalenian and the LA. The lithic assemblage consists of 4659 elements (including 3813 artifacts discovered during water screening). Around 50% of this assemblage was collected in US-102/103 while 3% was found in US-108 attributed to the Neolithic. The rest of the assemblage was collected in disturbed contexts. Considering technological and typological evidences, most of the assemblage discovered in disturbed context can, however, be related to the Azilian occupation. The lack of other Upper Paleolithic components on the site is fortunate.

The industry is primarily produced in flint (97.3%). It is important to point out that there are no flint outcrops in Brittany and more generally on the Armorican Massif. The first continental flint outcrops of the surrounding sedimentary basins are more than 300 km west of the site. In Brittany, flint is mostly available as pebbles on the banks of the Loire river that drained various formations of the Paris Basin, and, on the coast, as small pebbles transported from Cretaceous outcrops that today are located under the Channel. Most of the flint used at le Rocher de l’Impératrice is homogeneous and is very similar to coastal pebbles available on the shore of the Channel and Atlantic Ocean. The dimensions of some blades discovered at Le Rocher de l’Impératrice are however incompatible with the very small size of these pebbles. In addition, if a high proportion of artifacts shows cortex with evidence of erosion by submarine transport, some blades show remains of native cortex that suggest a collection of blocks directly from primary outcrops. The presence of such raw material is unique for the region and suggests that some of the actual submarine outcrops (the closest of which are ~100 km north of the site and today are under 90 m of water) were accessible during the EA occupation of le Rocher de l’Impératrice. The very few quartzite flakes were directly produced from the rock of the shelter while chalcedonic quartzite comes from a small deposit located 6 km Northeast of the site.

The technologic study of the currently available lithic assemblage shows an industry clearly oriented toward the production of blades (70% of the assemblage not including the water screening sample, and more than 89% of the tools). These blades are regular, mostly straight in profile, flat in cross-section and with acute cutting edges suggesting removal from cores with low transverse convexities. The average length of blades is 54 mm and the longest is 102 mm. The average width of the blades is 18.5 mm and average thickness is 5.2 mm. Blades can be divided in two distinct production objectives: larges blades (15 mm to 25 mm) produced and used unretouched or retouched in various types of tools, and narrower blades (12–13 mm) used to produce the projectile points. Bladelets were not a specific production objective. Just as flakes, they are only by-products of the blade reduction sequences.

**Fig 3:**
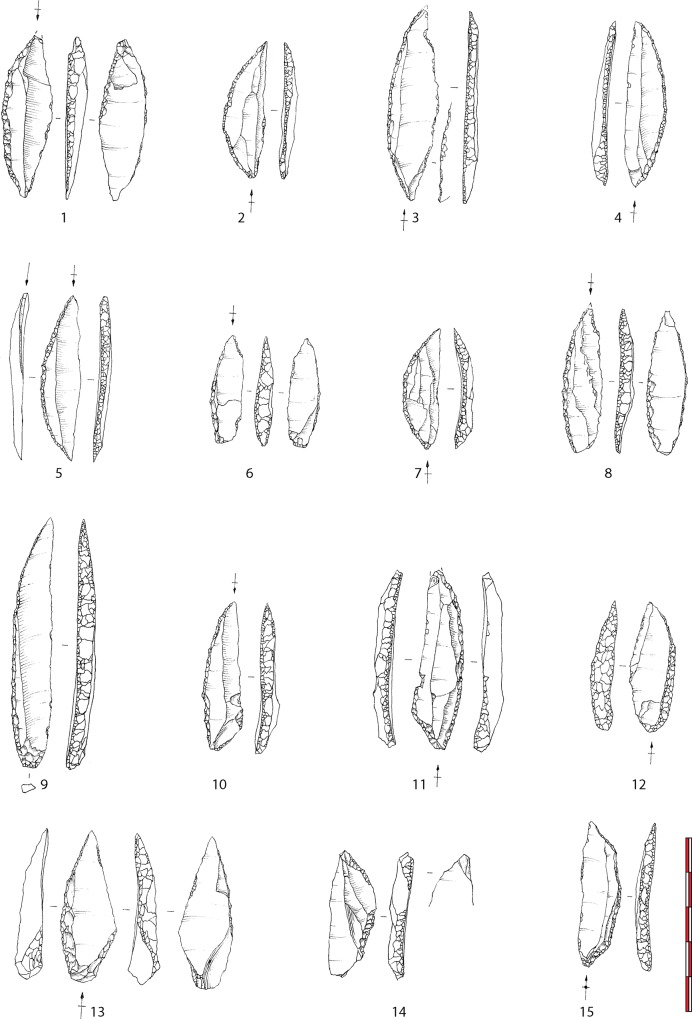
Projectile points from Le Rocher de l’Impératrice (drawing F. Blanchet). 1–8: bipoints; 9–11: regular monopoints (11: Grundy point); 12–13: irregular monopoints (13: Grundy point); 14–15: different stages of bipoints preforms.

The assemblage is particularly rich in lithic tools since 42% of the artifacts (11% with the screening) are retouched. Retouched tools are largely dominated by projectile points (54.4%) essentially composed of typical EA curved back bipoints (n = 25)(Fig 3). These bipoints are produced on small, regular and normalized blades (cv = 14% for width and 25% for thickness), and straight in profile. A few smaller bipoints seem to have been made from previously broken points. The retouch used to realize the backs of the bipoints is regular and produced by direct percussion with a hammerstone either on the left (50%) or right (50%) side of the blade. Except for the truncations used to shape the tips of the points, retouch is not invasive and the standardization of the point is mainly the result of the production of the blade itself and not of retouch. With these points are also curved back monopoints (n = 20). If typologically these armatures are common in LA assemblages, most of the monopoints of le Rocher de l’Impératrice were made with the same techniques and on the same regular blades as the bipoints. The co-occurrence of these two types is common during EA and has already been highlighted in other contemporaneous sites like Bois-Ragot [38], Pont d’Ambon [24] or La Fru [12]. Among these monopoints, some pieces are also realized on thicker and less regular blanks sometimes bearing a short line of retouches on the side opposed to the back on their bases. It is not clear yet if these points are evidences of a short occupation during the LA or if they are the illustration of a particular economic behavior in a region far away from raw materials.

**Fig 4:**
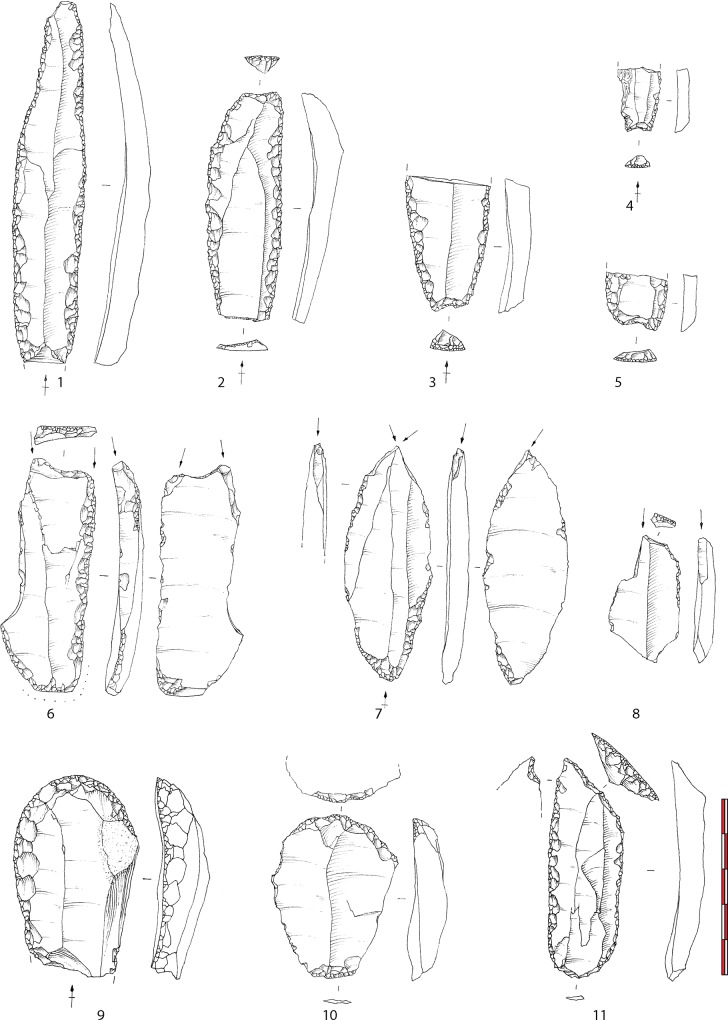
Domestic tools from Le Rocher de l’Impératrice (drawing F. Blanchet). 1–5: flat retouched blades; 6–8: burins; 9–10: end-scrapers; 11: borer.

Apart from these projectile points, tools (Fig 4) are represented by retouched blades (45.5% of the tools without the armatures). Most of these tools bear a particular flat retouch very common in EA assemblages [24, 38–39]. It is likely that this retouch was to maintain blades’ cutting edges as suggested by others [39]. Some of these retouched blades bear a characteristic concave truncation at one extremity that could have played a particular role in a hafting system. This combination has already been noticed by scholars in other EA sites [24, 38]. After being retouched, these blades were frequently recycled in other types of tools, essentially burins, but also end-scrapers and borers. Burins, mainly on truncations, are the second most represented tools after the retouched blades. End-scrapers are less represented and borers are rare. Contrary to end-scrapers or borers made on various types of blanks, frequently with cortical remains, often including byproducts of the blade production (crêtes, initialization blades), retouched blades and burins (that very frequently recycle retouched blades) are generally realized on the most regular blades of the industry. With the production of small blades used to produce Azilian curved back points, obtaining wider regular blades, probably used unretouched and maintained with a particular flat retouch, appears to be the central objective of production; other tools seem to be secondary at Le Rocher de l’Impératrice.

Because the assemblage is mainly composed of retouched tools and blades, it is difficult to discuss the methods used by EA groups to produce these tools. The technological study of the assemblage shows that blades were mainly obtained on cores with low transversal and longitudinal convexities after shaping of the core with crests. The systematic used of soft hammerstone direct percussion after meticulous abrasion of the edge of the platform helps reduction of this kind of surfaces. Two opposed platforms are frequently created. The use of the second platform is not systematic and the rhythm of alternation between the two platforms is not consistent.

These results show that, as in other EA site assemblages, that of le Rocher de l’Impératrice exhibits both characteristics inherited from the Magdalenian (importance and regularity of the blades, important role of burins in the production objectives) and those of the Azilian (systematization of the use of soft hammerstone and disappearance of soft hammers; disappearance of bladelet production; disappearance of backed bladelets—suggesting a possible disappearance of bone/lithic composite weapons, along with the development of axial back points). These results support other works conducted on EA assemblages suggesting a gradual dissolution of the Magdalenian concepts at the beginning of the EA [6, 11–12].

### 3.2. An exceptional graphic corpus

**Fig 5:**
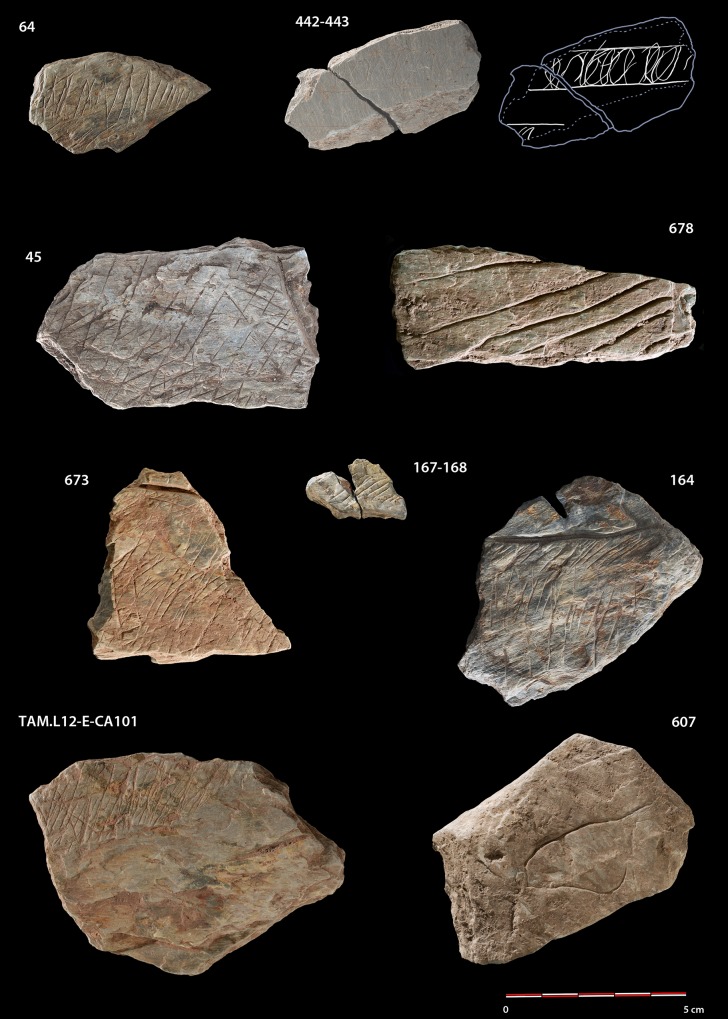
Selection of engraved pieces from Le Rocher de l'Impératrice (photos N. Naudinot; sketch C. Bourdier).

Le Rocher de l’Impératrice has provided 45 decorated stone pieces so far (S1 Table). With one exception (741), they all appear to be small, thin fragments of former larger slabs. Forty-three are less than 10 cm long, 29 of which are less than 5 cm. Three physical refittings have already been achieved (2 on Fig 5: fragments 167–168, and 442–443). All the blanks are local shales. The different shades and textures of the surfaces seem to separate seven potential engraved stones, that is, a small original corpus of decorated pieces went through a very high level of fragmentation (with one exception). Most of the fractures are old; some follow the lines of deep engravings. A few recent impacts are also evidenced. The pieces exhibit diverse processes and levels of taphonomic alteration with surfaces and tracings more or less patinated, and sediment deposits of different colors (yellow, ochre, black) and crusts. Such a diversity in the states of the natural surfaces can affect pieces from a single original slab as evidenced by the first refittings. Hence, the original number of decorated stones could be less than estimated. Most of the pieces are affected by an old flaking that differently affected the engravings, but none bears traces of rubefication.

**Fig 6:**
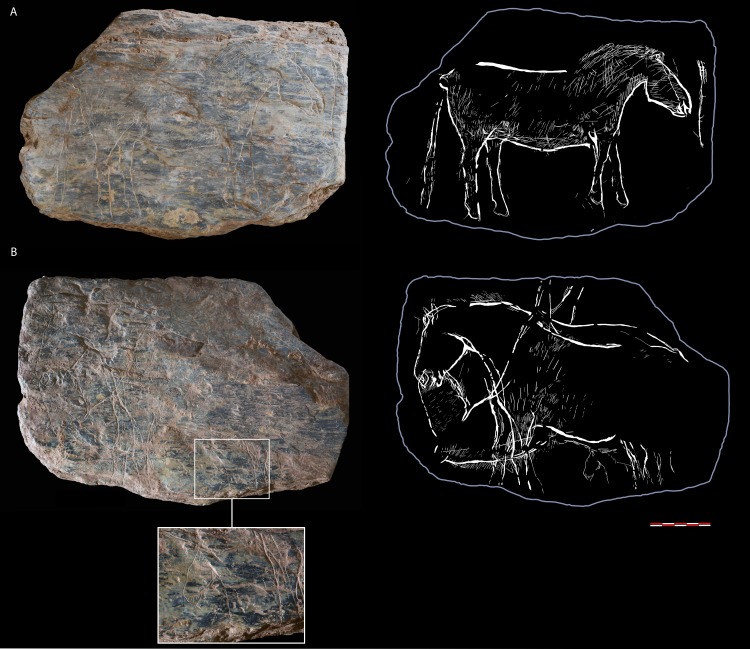
**Tablet 741 with bifacial ornamentation: side A) complete horse; side B) a special composition of two horses complete horses in axial symmetry–one complete and one headstock—and together with a small horse restricted to its head and neckprotomé by the hind legs (photos N. Naudinot; sketch C. Bourdier)**.

Considering the state of fragmentation, the selected blanks seem to be large stones as illustrated by the only complete piece of almost 30 cm long (Fig 6). Flat and smoothed surfaces were sought for etching. The general shapes of the blanks remain currently unknown excepted for the large rectangular slab 741 with rounded edges (Fig 6). The edge of fragment 64 was retouched to make it rounded. With regard to slab 741, this intervention could testify to a search of standardization in the shapes of the slabs. Marks of sawing on fragment 45 could have been intended to adjust an edge and to circumscribe the general frame of the setting. Such a framing could also be indicated on slab 741 face B on which a vertical line engraved in front of the horse seems to circumscribe the setting (Fig 6 - see below). Besides these two elements, no stone shows any evidence of pre-shaping or preparation. As documented in other sites such as Estebanvela [40], some pieces have a notch on their edge (Fig 5: fragment 164): their microscopic examination shows recent impacts within. Still their archaeological reality remains questioned as well as their possible function: pre-shaping? Fracturing initiation? No evidence of intentional fracturing has been observed so far. However, cleaning of the surfaces is necessary to confirm this first impression.

**Fig 7:**
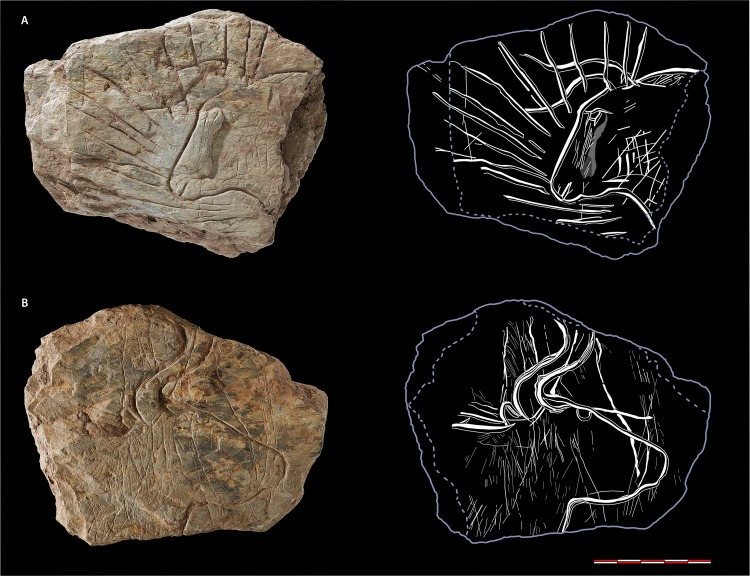
**Fragment 317 with a bifacial ornamentation: side A) head of aurochs surrounded by radiating lines; side B) head of aurochs (photos N. Naudinot; sketch C. Bourdier)**.

The ornamentation currently appears to be mainly unifacial even if the two largest pieces are engraved on their two opposite sides (Figs 6 & 7). However, since the fragments are thin, more back-to-back refits could produce more two-sided engravings in the future. Le Rocher de l’Impératrice graphic expressions are engravings of different types with the combination of wide and deep made by multiple passes and a variety of fine and superficial single tracings; surface abrasion is also documented on fragment 317. The fragments bear different densities of tracings. Some have several successive phases of engraving: fine and superficial lines are covered with wide and deep engravings. However, the fine and superficial engraving is still used to complete the inside of the motifs made in wide and deep incision. These additions and changes show that the tablets were reused and re-etched.

However, in the expectation of more refittings and the analytical tracing of the pieces, the motifs and formal conventions of these successive sets cannot be compared at present. Indeed, regarding the smallness of the pieces, the sets are difficult to decipher. Depictions of horses, aurochs, and geometric motifs–a triangle in a grid (Fig 5: fragment 45), a banner filled with loops (Fig 5: fragments 442–443)—are clearly identified. Organized sets of repeated simple shapes such as zigzags and chevrons could either be geometric patterns or fragments of depictions (Fig 5: fragments 64, 164, 673, TAM.L12-E-CA101—see below). However, other patches of lines show no apparent organization and appear to be the “unorganized tracings” well-known in the European Upper Paleolithic. A precise corpus of motifs is currently impossible to identify. Still, with regard to the estimated limited number of decorated tablets, the Le Rocher de l’Imperatrice collection of engravings strikes by the high number of animal depictions already identified. So far, the ornamentations are strictly monothematic.

A large slab depicts four of the five currently reported horses (Fig 6). Three subjects are grouped on one side: two are seen in mirror image in an unusual composition (741B). They fill the tablet and seem to use the edges as ground lines. They are represented full-length as is the isolated animal on the other side (741A). Included between the legs of one of the large horses is the head of a fourth horse. This position and the dissimilarity of size give the impression of a young foal between the legs of an adult, its juvenile appearance reinforced by its simplified design. The three other animals show a very similar rendering characterized by a strong naturalistic trend. The bodies are complete, well-proportioned, and shapely. The perspective inside the bodies also shows this desire to reflect reality with the depiction of the two legs–and the two ears on one subject–according to the same viewpoint. The lines of the legs are particularly fine on the isolated horse with the articulation of the wrist, the tip of the hock, the mention of ergot, pastern, hoof and heel at their extremity. The heads were given special attention. Particularly noticeable on the two subjects seen in mirror-view (741B), the shaping work reproduces the subtle rise at the start of the front, the convexity of the orbit, the slight depression of the forehead, the curve of the lips and the roundness of the jaw. Mouth and nostril are indicated but are not as precisely drawn. The absence of eye is a striking feature of these figures given the level of detail. One of the two horses seen in mirror also has two pointed triangular ears. Horse 741A shows special attention to the representation of the coat as a series of short oblique straight lines going along the outline of the animal from the chest to the chin groove, from the jaw to the muzzle, all along the forehead and in place of the mane reproduced by two parallel series. The hind leg and the belly are also covered with fine superficial engravings–vertical on the stifle and the buttock, oblique on the belly. The large horses on the other side bear these elements as well. The manes are also depicted similarly on the three animals as a series of short parallel tracings pointed towards. On the other hand, the small horse is represented in a much more schematic way. Made in a few cursory gestures, its outline is more rugged (steep ending of the muzzle for instance) and its anatomical details are only sketched (eye in single tracing, rectilinear ear). Its simple lines could match with the desire to draw a juvenile. Nevertheless, this very schematic rendering is found on another piece with the head of a horse bearing the same angular and pinched muzzle (Fig 5: fragment 607). More fine lines appear on side B but no motif has been deciphered out of them so far.

### 3.3. The “shining bull”: Special symbolic valorization of the aurochs?

Even if outnumbered by the horse engravings in the assemblage, the depiction of the aurochs seem to display a special emphasis. Two aurochs currently restricted to the heads are in opposition on the opposite sides of fragment 317 (Fig 7). They were outlined in wide and deep engraving with the search for an effect of *champlevé*. One side (A) bears a special composition of a bull’s head in left profile surrounded by deep rays that create a highlighting visual effect. No equivalent “shining animal” could be found in the European Paleolithic iconography. The technological study of this piece shows an intentional organization of gestures in order to point up the central place of the aurochs. The rays were engraved after the animal. But to place the aurochs at the forefront, the horns have been accentuated by a new series of engraving in the same grooves, occurring in the areas where the rays and the horns intersect.

In addition, the visual impact of the figure is enhanced by the coloring of the tracings with black pigment revealed by the cleansing of the incisions and evidenced by Raman spectrometry. Raman measurements were performed in the engravings at the black colorations and all recorded Raman spectra underline the presence of amorphous carbon (commonly called as a pigment “black carbon”). Even if black carbon is by nature poorly organized regarding its atomic structure, the Raman analyses could reveal a clear and specific structural signature (S1A Fig). Such result clearly excludes other possible black pigments such as manganese oxides which should be detected by Raman spectroscopy. However, the tablet is made of shale and such rocks are known to contain some carbonaceous materials [41]. Spectra were then recorded on the rock outside of the engravings and without macroscopically or microscopically noticeable black coloration; underlining that some amorphous carbon can be detected in the shale itself as illustrated in S1A Fig. However, by overlapping all the spectra from the pigment and the shale some slight differences could be observed in the spectral shape (S1B Fig). Even if it appears tiny, such differences are related to structural variations of the carbonaceous material according to their origin (carbonization or rock diagenetic process) [42], allowing the differentiation of the carbonaceous matter from various origins. The carbon signal detected in the engraving, therefore, does not originate from the shale itself. The carbon can only be explained by people introducing carbon onto the tablet, which is preserved in the engravings. Such enhancement of engraved motifs with color is rather unusual even if documented from time to time (some mobile art pieces from La Vache, Gourdan or Enlène for instance; [43–45]).

A very similar pattern of large and deep rays is found on another small fragment (Fig 5: fragments 167–168) suggesting that other shining figures could have been depicted. The “shining bull” may not be alone.

Besides this special visual valorization, the two animals exhibit a naturalistic rendering. Their outlines are shapely (convexity of the eye-socket, the chin and the lower jaw). The horns are sinuous. The tracings which diverge to their end would rather indicate two horns in single linear tracing seen in three-quarters. On side B, the two parallel tracings seem to belong to the same horn directed upward. The sensory organs are indicated but not as precisely drawn: round eye, mouth line. A nostril and a short pointed ear directly stuck in the outline at the back of the horn complete the aurochs of tablet 317B. The two subjects show some variations: the shaping work is more sensitive on the aurochs of 317B, and is also more detailed; the perspective applied to the horns also seems to be different. On the aurochs of 317A, a special attempt to reproduce the volume of the animal was made: the natural foliation of the shale was used and slightly retouched with scratching to suggest the relief of the zygomatic arch. The coat was given particular attention, reproduced in different ways: a series of parallel oblique short rectilinear lines stuck to the back line, a grid (317A) or patches of vertical and long superficial fine incisions at the base of the neck. Using 317A as a guide, other pieces could be fragments of animal silhouettes and/or internal fillings (Fig 5: fragments 46, 164, 46, 673, TAM.L12-E-CA101). Side B of 317 also bears numerous fine incisions of a former set made of animal depictions (herbivore forequarters?) covered in what currently appears as “unorganized tracings.”

## 4. Discussion

This collection testifies to formal and technical homogeneity, with some particular graphic conventions shared by different figures: the angular and pinched muzzles of the little horse on slab 741B and the horse on fragment 607, and the representation of the coat with a series of short oblique straight lines along the outlines of the horses on slab 741 and the aurochs on slab 317. Because of the obvious stylistic unity of these figures, we consider the corpus as a coherent assemblage in which coexist two formal renderings of the animal, one naturalistic, the other schematic. Even if most of these artifacts were discovered in the disturbed unit of the shelter, the entire corpus of engraved tablets can reasonably be attributed to the Early Azilian occupation(s) of Le Rocher de l’Impératrice as no evidence of occupation prior to the Azilian was found on the site.

**Fig 8:**
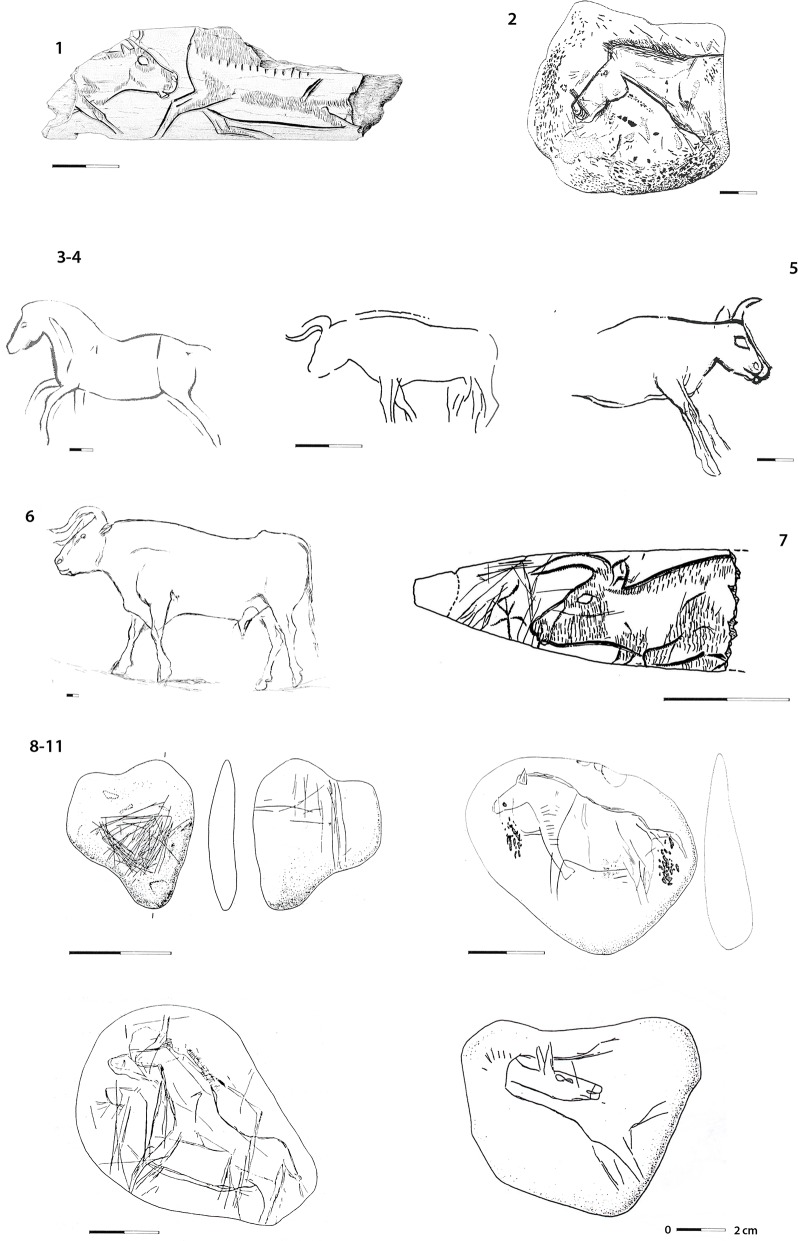
**Engraved mobile art from the Early Azilian (8–11) and the Upper/Final Magdalenian (1–7): 1) Le Morin (P. Laurent in [47]); 2) Villepin (G. Tosello in [48]); 3–4) La Madeleine (G. Tosello in [48]); 5) Limeuil (G. Tosello in [48]); 6) La Mairie cave (P. Paillet in [49]); 7) Rochereil (P. Paillet in [5]); 8–11) Murat rock-shelter (M. Lorblanchet in [25])**.

The 45 engraved stone fragments from Le Rocher de l’Impératrice are the oldest graphic remains in Brittany and prove to be exceptional for the EA. In France, less than a dozen sites have yielded decorated items related to this techno-complex, most of them from uncertain contexts. Among them, only three collections of mobile art can be clearly attributed to this culture: the 82 engraved lithic artifacts of Murat rock-shelter (layers B and D of Lemozi, layer IV of Lorblanchet: [25]), the five flakes with engraved cortex of Le Closeau [14], an engraved pebble of Bois-Ragot (layer 4: [26]). A single parietal art site is attributed to the EA- Gouy cave [23, 46]–on the basis of the lithic industry found at the site; however, no direct link exists so far between the parietal set and the archaeological deposit. In this regard, the collection of Le Rocher de l’Impératrice considerably increases the data. Even if it is difficult to draw general conclusions about the Early Azilian symbolic system from these few sites, and the very restricted corpus for Le Closeau and Le Bois-Ragot, a coherent image of these graphic works still appears: the notable selection of small pebbles and slabs as blanks, and the mostly unifacial sets made of a unique motif or a very small number of superimposed motifs. The iconography combines both geometric themes such as checkered triangles, lines, or beams, and figurations with bestiaries dominated by horses and aurochs (Fig 8). The schematic animals depicted in Gouy cave bear stylized coats in the form of grid patterns or parallel lines reminiscent of the aurochs on tablet 317 [46], together with angular and pinched muzzles for the horses. Murat rock-shelter figures also show two types of formal rendering: schematic representations cohabit with realistically shaped and dynamic animals with simplified internal details (Fig 8).

The EA iconography of Le Rocher de l’Impératrice is very similar to the Upper-Final Magdalenian and several elements suggest direct continuity. That is the case of the selected materials to be engraved: pebbles and tablets are also abundantly used in large lithic mobile art assemblages such as Limeuil, Gourdan, Gönnersdorf, La Peña de Estebanvela and Foz do Metal [40, 48, 50–53]. This similarity can also be seen in the technical preference for engraving instead of painting. The compositions associating geometric patterns, figures and unorganized tracings is another common pattern. The themes are also very similar: the geometric register appears to remain rather unchanged with the predominance of chevrons, grids, lines beams, together with triangles and ellipses. As to the figurative register, aurochs and horses also fill the Upper/Final Magdalenian bestiary even if aurochs are not very common. The formal conventions of the animals in Le Rocher de l’Impératrice match well with those documented of the Upper-Final Magdalenian: a realistic rendering with complete bodies, with the two finely reproduced pairs of legs, seen in real perspective, shapely silhouettes, and numerous anatomical details depicting the sensorial organs, the internal volumes and the coat. Macrocephaly of the horses is another strong figurative trend [5, 54]. These formal traits are shared within the whole graphic production of the second half of the Magdalenian in Western Europe, from the Paris Basin with the engraved pebble from Étiolles [55] to the North of Spain (Abauntz: [56]; in others). They are also the ones of some parietal paintings directly dated between 13,500 and 12,500 BP at Niaux [57], La Pasiega C [58], and El Castillo [58]. Some particular Middle-Upper Magdalenian conventions can be more specifically pointed: the depiction of the zygomatic depression (aurochs 317A, horse 741A?), the stylized representation of the coat as a series of short, straight oblique lines stuck to the back line (aurochs on fragment 317, horse 741A) or as a fine hatching filling inside the legs and the belly (horses on tablet 741) [5, 44, 48–49, 51, 59]. A less detailed formal rendering could be argued for the engravings at Le Rocher de l’Impératrice but simplified representations are also found in the Upper-Final Magdalenian together with very naturalistic figures, some turning very schematic, sometimes within the same assemblage (e.g. abri Mège, La Mairie, Le Morin, Limeuil, Laugerie-Basse, Rochereil or La Madeleine–[49, 60]).

From these results we suggest continuity in the iconographic tradition between the Upper Magdalenian and the EA, as already suggested by M. Lorblanchet in Murat [25]. The dearth of artistic items from the EA nevertheless limits our estimation of the degree of longevity after the Upper Magdalenian. However, the special valorization of the aurochs as shown by the “shining bull” could indicate some symbolic recoding of the traditional bestiary. Evidence is too scarce at the moment to develop this hypothesis further. We also suggest that the chrono-cultural attribution of some Upper Magdalenian mobile art collections should be reevaluated with the possibility that some may be more recent since many assemblages were actually collected in sites where the Upper Magdalenian layers are generally covered with EA and LA layers (e.g., La Madeleine, Villepin, grotte Richard; [48]; Laugerie-Basse: [61]). However, this continuity is particularly striking with regard to the Southern cultural context of Northern Spain where the Upper-Final Magdalenian lasts until 11,800–11,600 BP whereas the EA develops in France [62]. Thus, two neighboring cultural groups with different domestic and hunting equipment but a common iconography seem to coexist (i.e., in others La Vache, Le Portel, Ekain VIb, Oscura de Ania IIIa, Abauntz 2r, Las Monedas, La Pasiega C–[44, 63–66]).

These results provide the first picture of a graphic tradition in the EA clearly distinctive from the LA whose iconography is dominated by painted and engraved abstract patterns on pebbles; however such abstract pebbles could be already present in the EA in Murat rock-shelter (layers B and D of Lemozi, layer IV of Lorblanchet). Similar pebbles in Bois-Ragot (layer 4), Gay rock-shelter and Rochedane [2, 67], are likely from mixed contexts containing EA and LA materials. Where abstract pebbles are found in secure contexts, there is a clear iconographic schism between the EA and LA in regard to 1) a shift in material selection with use only of standardized pebbles, 2) painting as a preferred technique, 3) the explosion of abstract expressions in the shape of repeated simple patterns (points, lines, crosses, chevrons), 4) the correlative scarcity of depiction whether animal or human, and 5) a change in the formal rendering of the figures in favor of geometric schematism. The lines are simplified and rugged (triangular legs without hoof), the bodies are wide and the heads atrophied. Most often, the internal details are missing. The bodies can be blank or filled with patches of long tracings, oblique or horizontal on the flank, vertical in the legs (Murat: [25]). Similar iconographic changes accompany the spread of the classical Azilian in the North of the Iberian peninsula [53, 68].

**Fig 9:**
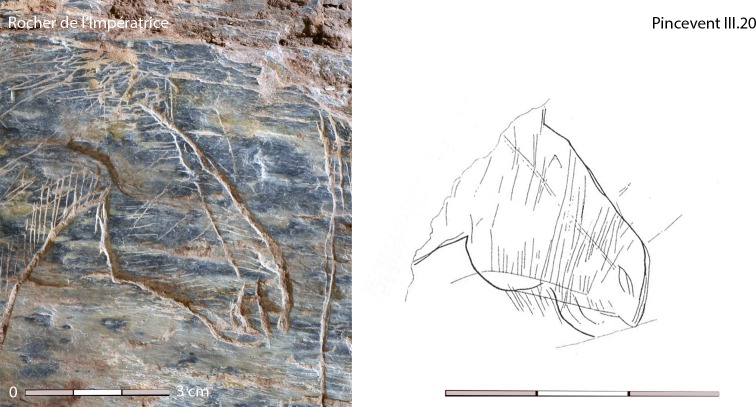
Similarities of conventions in the formal rendering of the horse heads from Le Rocher de l'Impératrice and Pincevent (layer III.20) (photo N. Naudinot; tracing D. Baffier in [69]).

The data presented here highlight a discrepancy between technical and symbolic changes during the azilianization process. If the graphic corpus of Le Rocher de l’Impératrice testifies to iconographic continuity with the Magdalenian, the lithic assemblage already bears all the Azilian characteristics with especially the exclusive use of soft hammer stones and the exclusive use of axial projectile points to the detriment of backed bladelets. The major transformation of the symbolic system with the development of a geometric expression and the correlative near-abandonment of animal and human figures seems to start later. While the data are still too scarce to determine precisely when a shift in symbolic system begins, those available data suggest the beginning of the Allerød. The move away from Magdalenian and EA iconography probably occurred after the beginning of the LA. This timing is suggested by the discovery of a horse head still bearing the EA formal conventions (arched nose, pinched lip, few details but the mention of the zygomatic depression and the coat represented by series of long vertical lines) engraved on cortex found in layer III.20 at Pincevent dated around 13,600 cal. BP at the beginning of the LA ([69–70]; Fig 9).

This study underlines a difference in rhythm between technological and symbolic changes of the Magdalenian and EA. Our data suggest a progressive dissolution of the Magdalenian lithic standards while conventions of Magdalenian iconography seem to change later and abruptly by the Allerød. The likely disappearance of animal and human figures in favor of an exclusive abstract expression is a crucial iconographic shift with regards to the other former Upper Palaeolithic societies and to the subsequent final Paleolithic populations in Western Europe. While it is possible that this picture of a clear scission in the symbolic sphere is only caused by the lack of data between EA and LA, the findings at Le Rocher de l’Impératrice suggest that Magdalenian iconography is greatly extend in time.

The onset of the Azilian is not a package of changes affecting both the symbolic system and technical equipment. It is essential to understand the origin of this critical evolution in symbolic expression, which may bear important ideological and sociological significance during the Azilian. The simple techniques, motifs, as well as the large residential sites in which the decorated pebbles are generally unearthed would rather indicate common productions of these items in the LA. This marks an important shift from Magdalenian graphic production [62], which emphasizes a complex technical *savoir-faire*, realism, and a degree of detail in numerous representations that are without any doubt the work of specialists who went through a distinct learning process [71–72]. According to Gonzalez Morales [73], the abandonment of figures for geometric expression would be linked to changes in the techniques used and in the actors, and the three would result from a complete series of global changes in the social organization of graphic production. In parallel, the decrease in *savoir-faire* can also be identified in the lithic and bone technology of the LA. It is tempting to see these phenomena as a possible development of a common production strategy that minimized the importance of specialists in both graphic and technical activities. These data could suggest a major sociological shift during the Allerød. Changes in mobility patterns, judged by the few data available, suggest a potential increase in residential mobility during the LA [6, 13, 74–76]. This change in mobility is important to consider as the reorganization of activities in time and space may be profoundly linked with these cultural changes.

In human cultural evolution, changes in the cultural system do not necessarily affect all elements of material culture at the same time [62, 77]. This is sometimes contrary to the general idea conveyed in archaeological studies. This vision is mostly perpetuated by the nature of the archaeological record as our theories and interpretations are often based on an extremely fragmentary subset of remains from past cultural systems. Sites like Le Rocher de l’Impératrice with remains of various parts of the cultural system allow us to discuss and compare differences in the tempo of cultural change. In this case, the development of Azilian cultural or symbolic acts appear to lag behind major technological adaptations.

## Supporting information

S1 Table Engraved tablets database. Captions: L/D: Large and deep engraving; F/S: Fine and superficial engraving; F.D: Fine and deep engraving; W/D: Wide and deep engraving; W/D+Ch: Wide and very deep engraving with champlevé; SPSC: Small patches of sediment crusts; SPSD: Small patches of sediment deposit; SPSDC: Small patches of sediment deposit and crusts; HP: Highly patinated surface; HEE: Highly eroded edges; HPT: Highly patinated tracing; OAA: Other anthropological action; 1: Blue grey; 2: Visible lamination; 3: metallic light grey; 4: light and dark blue; 5: Shiny lamination; 6: Pearlescent pinkish light grey; 7: Matt slate grey; 8: Matt dark blue grey; 9: Grey
(DOCX)

S1 Fig a)Several Raman spectrum obtained on the black pigment in the engraving compared with some obtained on the shale tablet out of engraved areas and without visible black colour. These signatures are constituted by the symmetric stretching of carbon-carbon single bonds (band around 1367 cm^-1^) and carbon-carbon double bonds (band around 1597 cm^-1^). Spectra are baseline corrected to remove the fluorescence contribution. Several spectra from the two kind of areas are overlapped to underline reproducibility, and the two vertically shifted for readability. b) Same spectra from both areas overlapped in order to highlight the shapes different between the pigment and the carbonaceous matter from the shale.
(DOCX)
