# Correction: Divergence in the evolution of Paleolithic symbolic and technological systems: The shining bull and engraved tablets of Rocher de l’Impératrice

**DOI:** 10.1371/journal.pone.0204464

**Published:** 2018-09-18

**Authors:** Nicolas Naudinot, Camille Bourdier, Marine Laforge, Céline Paris, Ludovic Bellot-Gurlet, Sylvie Beyries, Isabelle Thery-Parisot, Michel Le Goffic

There is an error in Table 2. The values in column 5 "2 sigma calibration (cal. BP)" are incorrectly listed in cal. BC, not cal. BP. Please see the corrected Table 2 here.

**Table 2:** Radiocarbon dates for the French Early Azilian.

Site	Layer	Laboratory code	Conventional Radiocarbon age	2 sigma calibration (cal. BP)	Reference	Remarks
Pont d’Ambon	4 sup.	Gif-3369	12840 ± 220	16017 to 14405	[24]	
Bois-Ragot	4	OxA-10334	12720 ± 100	15531 to 14717	[30]	
Abri Murat	4	GifA-92345	12620 ± 130	15341 to 14260	[31]	
Bois-Ragot	4	OxA-10333	12585 ± 75	15210 to 14447	[30]	
Le Closeau, locus 33	Intermediary	GrA-18860	12510 ± 80	15107 to 14275	[32]	
Le Closeau, locus 33	Intermediary	GrA-18815	12480 ± 70	15058 to 14252	[32]	
Bois-Ragot	4	OxA-10332	12475 ± 75	15059 to 14236	[30]	*Might be from the Magdalenian layer*. *Date on a reindeer bone*
Pont d’Ambon	3B	Ly-6433	12450 ± 70	15006 to 14205	[13]	
Abri Murat	4	Poz-27957	12430 ± 80	14992 to 14164	[13]	
Le Closeau, locus 46	Lower layer	AA-41881	12423 ± 67	14943 to 14169	[32]	
Le Closeau, locus 46	Lower layer	GrA-11665	12360 ± 90	14905 to 14070	[32]	
Le Closeau lower layer locus 46	Lower layer	GrA-18816	12350 ± 70	14784 to 14082	[32]	
Le Closeau lower layer locus 46	Lower layer	GrA-11664	12350 ± 60	14740 to 14098	[32]	
Le Closeau lower layer locus 56	Lower layer	GrA-18819	12340 ± 70	14763 to 14074	[32]	
Abri Murat	4	Poz-27958	12330 ± 80	14790 to 14044	[13]	
La Fru, aire 2	3		12300 ± 70	14690 to 14030	[33]	
Rhodes II	F5	MC-996	12300 ± 150	14994 to 13830	[34]	
La Fru, aire 2	3		12250 ± 60	14495 to 13967	[33]	
Rhodes II	F5	MC-1366	12250 ± 200	15075 to 13740	[34]	
Le Closeau, locus 46	Lower layer	AA-41882	12248 ± 66	14547 to 13952	[32]	
La Fru, aire I	3	Gr1-34354	12200 ± 50	14260 to 13935	[33]	
Rhodes II	F5	Gif-2258	12160 ± 160	14786 to 13610	[34]	
La Fru, aire I	3	Ly-134/Oxa-5264	12110 ± 110	14362 to 13713	[33]	
Pont d’Ambon	3B base	Gif-3739	12130 ± 160	14706 to 13582	[24]	
Rhodes II	F6 base	MC-997	12100 ± 150	14588 to 13570	[34]	
Le Closeau, locus 4	Lower layer	OxA-5680 (Lyon 166)	12090 ± 90	14172 to 13745	[14]	
Bange	E/G	OxA-540	12080 ± 180	14710 to 13490	[33]	
Gouy		GifA-92346	12050 ± 130	14264 to 13563	[35]	
Le Closeau, locus 4	Lower layer	OxA-6338 (Lyon 313)	12050 ± 100	14173 to 13630	[14]	
La Fru, aire I	3 base	GrA-25052	11950 ± 60	13987 to 13589	[33]	
Les Douattes, Est	4/5, F5-64	Ly-1417	11945 ± 85	14021 to 13566	[33]	
St-Thibaud-de-Couz		Ly-429	11900 ± 360	15062 to 13089	[33]	
Balma Margineda	10	Ly-4898	118701 ± 110	13984 to 13466	[36]	
La Fru, aire I	3	GrA-25080	11840 ± 60	13777 to 13489	[33]	
La Fru, aire I	3	Ly-2408	11820 ± 230	14290 to 13137	[33]	
La Fru, aire I	3	Ly-2250	11810 ± 160	14034 to 13322	[33]	
La Fru, aire I	upper 3	GrA-25054	11790 ± 60	13746 to 13477	[33]	
La Fru, aire 1	3 base	Ly-4325	11740 ± 110	13778 to 13339	[33]	
Balma Margineda	10	Ly-4896	11690 ± 90	13735 to 13342	[36]	
La Fru, aire I	3 base	Ly-2409	11680 ± 150	13820 to 13189	[33]	
Hangest III.1	Lower layer	OxA-4432 (Ly-22)	11660 ± 110	13734 to 13289	[37]	Probably older. Layer under the Allerød soil
Bois-Ragot	4	Lyon-2754 (OxA)	11640 ± 55	13579 to 13347	[30]	
Hangest III.1	Lower layer	OxA-4936 (Ly-86)	11630 ± 90	13709 to 13275	[37]	Probably older. Layer under the Allerød soil
Balma Margineda	9	Ly-5416	11600 ± 280	14111 to 12851	[36]	
Pont d’Ambon	3B base	Gif-7223	11600 ± 120	13717 to 13206	[24]	
Balma Margineda	10	Ly-5413	11560 ± 230	13967 to 12987	[36]	
Balma Margineda	10	Ly-5414	11510 ± 100	13547 to 13143	[36]	
Balma Margineda	10b	Ly-5415	11500 ± 150	13701 to 13065	[36]	

## References

[1] Naudinot N, Bourdier C, Laforge M, Paris C, Bellot-Gurlet L, Beyries S, et al (2017) Divergence in the evolution of Paleolithic symbolic and technological systems: The shining bull and engraved tablets of Rocher de l’Impératrice. PLoS ONE 12(3): e0173037 10.1371/journal.pone.0173037 28257445 PMC5336238

