# Supplementary material for: Divergence in the evolution of Paleolithic symbolic and technological systems: The shining bull and engraved tablets of Rocher de l'Impératrice
Source: PLoS One. 2017 Mar 3;12(3):e0173037. doi: 10.1371/journal.pone.0173037 (PMC5336238; doi:10.1371/journal.pone.0173037)
Supplement: S1 Table — Captions: L/D: Large and deep engraving; F/S: Fine and superficial engraving; F.D: Fine and deep engraving; W/D: Wide and deep engraving; W/D+Ch: Wide and very deep engraving with champlevé; SPSC: Small patches of sediment crusts; SPSD: Small patches of sediment deposit; SPSDC: Small patches of sediment deposit and crusts; HP: Highly patinated surface; HEE: Highly eroded edges; HPT: Highly patinated tracing; OAA: Other anthropological action; 1: Blue grey; 2: Visible lamination; 3: metallic light grey; 4: light and dark blue; 5: Shiny lamination; 6: Pearlescent pinkish light grey; 7: Matt slate grey; 8: Matt dark blue grey; 9: Grey (DOCX) [file pone.0173037.s001.docx]

| **N°** | **Square** | **L** | **W** | **T** | **Color** | **Completeness** | **Taphonomy** | **Techniques** | **Iconography** |
| --- | --- | --- | --- | --- | --- | --- | --- | --- | --- |
| 45 | L11 |  |  |  |  |  |  |  |  |
| 64 | M11 | 51 | 28 | 5 | 1+2 | Frg | 3 fract edge/Patches of reddish crust | F/D-W/D | Parallel short rectilinear tracings (coat? Mane?)/OAA: Rounded edge |
| 70 | M11 | 42 | 33 | 5 | 1+2 | Frg | 2 fract edge/Patches of reddish crust | F/D-W/D | A simple line and a curve |
| 91 | L11 | 44 | 28 | 4 | 3 | Frg | 4 fract edge/Patches of sediment deposit | W/D | Single undulating line |
| 163 | P13 | 33 | 18 | 3 | 4+2 | Superf. Spall | 4 fract edge (2 recent)/Small patches of black crust | F/S-F/D-W/D | High density of superimposed rectilinear and curved lines |
| 167 | M12 | 22 | 13 | 3,6 | 1+5 | Frg. Refits 168 | 4 fract edge (3 recent)/1 fract along a E: W/D | F/S-W/D | Parallel curves lines (radiating motif?) |
| 168 | M12 | 18 | 10 | 4 | 3+5 | Superf. Spall refits 167 | 4 fract edge (1 recent)/1 fract. Along E: W/D | F/S-F/D | End of 3 parallel rectilinear lines (coat?) |
| 210 | O13 | 55 | 41 | 6 | 6 |  | 4 fract edge/Strong flak left side/Patinated tracings | F/S-F/D | Fragment of silhouette with internal filling in superficial engraving |
| 262 | M12 | 49 | 15 | 3 | 1+5 |  | 2 fract edge (1 recent)/ 1 natural face | F/S-F/D-W/D | Fragment of depiction |
| 268 | O13 | 64 | 37 | 13 | 4 |  | SPSD | F/S | 2 long parallel rectilinear lines |
| 317 | N12 | 128 | 102 | 16 | 1 | Frg. Refits 678 and 673 | 1 natural edge/3 frat edge/Smooth+depict/flacking | F/S-F/D-W/D-W/D^+^CH-BP | 1 head of aurochs on each side; fragments of animal depiction; unorganized tracings/OAA Intentional impact on a lateral edge? |
| 442 | L14 | 42 | 28 | 3 | 7 | Frg. Refits 443 |  | F/S-F/D | Pattern made of 2 banners filled with loops |
| 443 | L14 | 30 | 20 | 3 | 7 | Frg. Refits 442 |  | F/S-F/D |  |
| 607 | N13 | 79 | 51 | 13 | 7 | Frg. | 2 fract edge/1 nat edge/superf flack along edges | F/S-D/D-W/D | Horse head |
| GRA1 | L14 | 40 | 23 | 9 | 6 | Frg. | 4 fract edge/superf flack | F/S | Series of parallel rectilinear tracings (coat?) |
| GRA10 | N13 | 22 | 19 | 4 | 6 | Frg. | 2 fract edge/Small spatches sedim. Deposits | F/S | End of 2 rectilinear lines |
| GRA11 | L15 | 20 | 9 | 2 | 1+5 | Frg. | 4 fract edge/Highly patinated tracing/sediment crusts | F/S | High density of apparently unorganized tracings |
| GRA12 | L15 | 19 | 11 | 2 | 1+5 | Frg. | 4 fract edge/Highly patinated tracing/sediment crusts | F/S | High density of apparently unorganized tracings |
| GRA13 | L15 | 17 | 15 | 3 | 1 | Frg. | 4 fract edge | F/D | End of line/OAA: intentional impact on a lateral edge? |
| GRA14 | L16 | 19 | 11 | 3 | 1 | Frg. | 3 fract edge/1 nat edge/patinated surf/Patches black low face | F/D | Ends of lines |
| GRA15 | L16 | 21 | 15 | 4 | 1 | Frg | All fract edge/HPS | F/S | 2 series of rectilinear tracings |
| GRA16 | L16 | 22 | 15 | 6 | 1 | Frg. | 3 fract edge/Patinated surf | F/D | End of line |
| GRA17 | L15 | 17 | 13 | 5 | 1 | Frg. | 2 fract edge/1 natural edge | F/S | End of line |
| GRA18 | L15 | 25 | 20 | 2 | 3 | Superf. Spall | 4 fract edg1 recent/Patinated surf/SPSD | F/S-F/D | Patches of tracings in diverse orientations (silhouette and internal fillings?) |
| GRA19 | L15 | 76 | 58 | 13 | 4 | Frg. | 3 fract edge/1 nat edge/patinated surf/Patches black low face | F/S-F/D | Few lines including a curve associated with a series of short oblique straight lines (fragment of depiction?) |
| GRA2 | N13 | 37 | 35 | 7 | 6 | Superf. Spall | 3 fract edge/1 nat edge/patinated surf/superf flak edge/SPSC | F/D-W/D | End of lines |
| GRA3 | N13 | 24 | 22 | 2 | 6 | Superf. Spall | All fract edge/HPS/SPSDC | F/S-F/D-W/D | Patches of tracings in diverse orientations (silhouette and internal fillings?) |
| GRA4 | N13 | 45 | 42 | 10 | 6 | Frg. | All fract edge/HPS/SPSDC | W/D | Fragment of depiction (ear and oval eye)? |
| GRA5 | N13 | 42 | 34 | 8 | 6 | Frg | All fract edge/HPS/Superficial old flaking | W/D | Fragment of depiction (neck and mandible)? |
| GRA6 | N13 | 41 | 24 | 5 | 6 | Frg. | 4 fract edge/HPS/Superficial old flaking/SPSDC | F/S-F/D-W/D | Series of converging long lines |
| GRA7 | N13 | 35 | 25 | 2 | 6 | Frg. | 4 fract edge/HPS/Superficial old flaking/SPSDC | F/S-F/D-W/D | Patches of tracings in diverse orientations (silhouette and internal fillings?) |
| GRA8 | N13 | 52 | 30 | 6 | 6 | Frg. | 1 fract edg/superf flak/patinated surf/superf black deposit | F/S | End of tracing |
| GRA9 | N13 | 30 | 13 | 2 | 6 | Superf. Spall | 3 fract edge/1 nat edge/superf flak/patinated surf/SPSC | F/D | 2 converging tracings |
| GRA20 | N13 | 35 | 21 | 4 | 6 | Superf. Spall | All fract edge/superf flak/ocher crust | F/S-F/D | Curve |
| GRA21 | N12 | 21 | 13 | 4 | 1+2 | Frg. 2ref pieces | All fract edge/pat surf/Sed dep+crust in tracing/SPSD | F/S-F/D-W/D | Fragment of depiction (coat and eye)? |
| GRA22 | N12 | 27 | 24 | 4 | 1 | Frg. | 4 fract edge/HEE/HPS/SPSD+engrav | F/S-F/D-W/D+-CH | Fragment of silhouette with internal filling in superficial engraving |
| GRA23 | M11 | 20 | 10 | 4 | 1 | Frg. | 4 fract edge/1fract WD tracing/pat surf/small patch ochred | F/D-W/D | Fragment of silhouette with internal filling in superficial engraving |
| 673 | N13 | 55 | 56 | 5 | 1+2 | Frg. Refits 317 | 3 fract edge/HPT/SPSC | F/S-F/D-W/D-W/D+CH | Fragment of silhouette with internal filling in superficial engraving (neck/back line?) |
| 678 | N13 | 86 | 35 | 6 | 9+2 | Frg. Refits 317 | 4 fract edge/pat surf/SPSC surf+engrav | F/S-W/D+CH | Radiating lines |
| 695 | N13 | 63 | 42 | 7 | 6 | Frg. | All fract edge/SPSC surf+engrav | F/S-F/D-W/D | Patches of tracings in diverse orientations (silhouette and internal fillings?) |
| 696A | N13 | 97 | 75 | 14-9 | 6 | Frg. | All fract edge 1 recent/flak/Pat concavities/SPSC surf+engrav | F/S-F/D-W/D | Patches of tracings in diverse orientations, some organized (silhouette and internal fillings?), others apparently unorganized |
| 696B | N13 | 88 | 51 | 4 | 6 | Frg. | All fract edge 1 recent/flak/pat concavities/SPSD surf+engrav | F/S-F/D-W/D | 2 series of long curved lines (fragment of depiction?) |
| 741 | O12 | 272 | 189 | 36 | 7 | Entire | Area pat surf/SPSC and within the engraving |  | (A) 2 complete horses, a horse head, and apparently unorganized tracings;(B) 1 horse; |
| GRA24 | O12 | 24 | 11 | 2 | 1+2 | Superf. Spall | All fract edge/Small patches of ochred crust | F/S-W/D | 2 parallel slightly curved lines (legs?) |
