# Supplementary material for: Divergence in the evolution of Paleolithic symbolic and technological systems: The shining bull and engraved tablets of Rocher de l'Impératrice
Source: PLoS One. 2017 Mar 3;12(3):e0173037. doi: 10.1371/journal.pone.0173037 (PMC5336238; doi:10.1371/journal.pone.0173037)
Supplement: Supplementary file 2 [file pone.0173037.s002.docx]

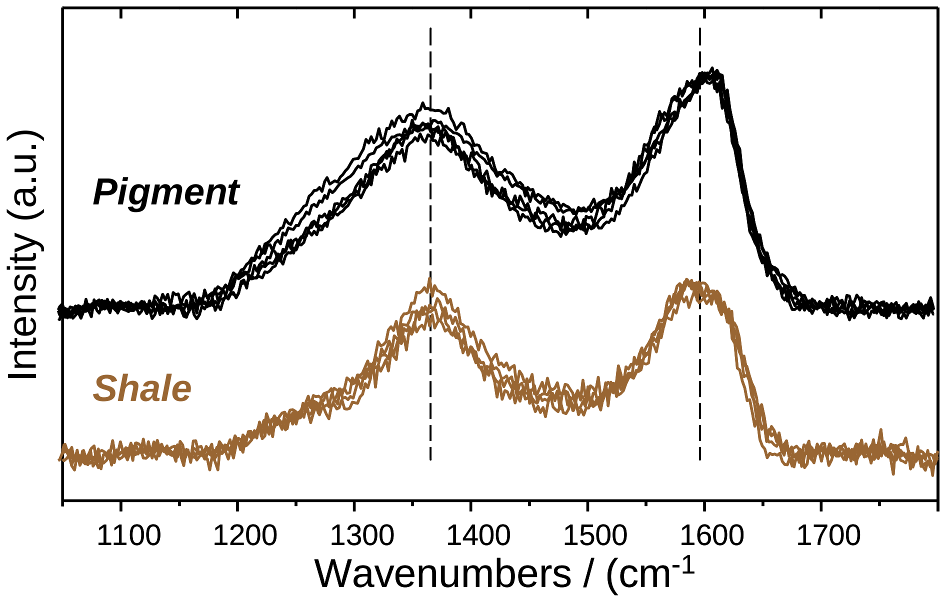
Pigment
Shale
Intensity (a.u.)
1100
1200
1300
1400
1500
1600
1700
Wavenumbers / (cm-1

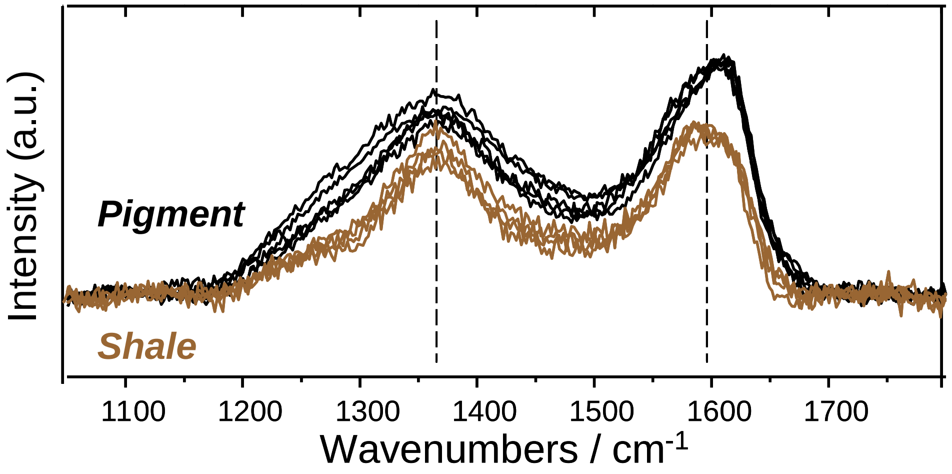
Pigment
Shale
Intensity (a.u.)
1100
1200
1300
1400
1500
1600
1700
Wavenumbers / cm-1
